# Intrathecal Delivery of IL-6 Reactivates the Intrinsic Growth Capacity of Pyramidal Cells in the Sensorimotor Cortex after Spinal Cord Injury

**DOI:** 10.1371/journal.pone.0127772

**Published:** 2015-05-19

**Authors:** Ping Yang, Yu Qin, Chen Bian, Yandong Zhao, Wen Zhang

**Affiliations:** 1 Department of Neurobiology, Chongqing Key Laboratory of Neurobiology, Third Military Medical University, Chongqing, 400038, P.R China; 2 Cadet Brigade, Third Military Medical University, Chongqing, 400038, P.R China; Hertie Institute for Clinical Brain Research, University of Tuebingen., GERMANY

## Abstract

We have previously demonstrated the growth-promoting effect of intrathecal delivery of recombinant rat IL-6 immediately after corticospinal tract (CST) injury. Our present study aims to further clarify whether intrathecal delivery of IL-6 after CST injury could reactivate the intrinsic growth capacity of pyramidal cells in the sensorimotor cortex which project long axons to the spinal cord. We examined, by ELISA, levels of cyclic adenosine monophosphate (cAMP), adenylyl cyclase (AC, which synthesizes cAMP), phosphodiesterases (PDE, which degrades cAMP), and, by RT-PCR, the expression of regeneration-associated genes in the rat sensorimotor cortex after intrathecal delivery of IL-6 for 7 days, started immediately after CST injury. Furthermore, we injected retrograde neuronal tracer Fluorogold (FG) to the spinal cord to label pyramidal cells in the sensorimotor cortex, layers V and VI, combined with βIII-tubulin immunostaining, then we analyzed by immunohistochemisty and western blot the expression of the co-receptor gp-130 of IL-6 family, and pSTAT3 and mTOR, downstream IL-6/JAK/STAT3 and PI3K/AKT/mTOR signaling pathways respectively. We showed that intrathecal delivery of IL-6 elevated cAMP level and upregulated the expression of regeneration-associated genes including GAP-43, SPRR1A, CAP-23 and JUN-B, and the expression of pSTAT3 and mTOR in pyramidal cells of the sensorimotor cortex. In contrast, AG490, an inhibitor of JAK, partially blocked these effects of IL-6. All these results indicate that intrathecal delivery of IL-6 immediately after spinal cord injury can reactivate the intrinsic growth capacity of pyramidal cells in the sensorimotor cortex and these effects of IL-6 were partially JAK/STAT3-dependent.

## Introduction

In the adult central nervous system (CNS), axons are usually not able to regenerate after lesions, while axons in the CNS during the first postnatal week and axons in the peripheral nervous system (PNS) do. It has been proposed that declined intracellular cAMP level during development [1] is one of the main reasons for the failure of the CNS regeneration [2–3]. The spontaneous age-dependent decrease of cAMP in neurons not only mediates the switch from promotion to inhibition of axonal elongation on myelin substrate but also results in the loss of neuronal intrinsic growth capacity. After spinal cord injury, levels of cAMP in neurons of the brainstem and sensorimotor cortex decrease substantially, especially in their injured axon ends. Up-regulation of cAMP has been shown to promote axonal regeneration and functional recovery [4].

Park and Liu et al found that PTEN/mTOR signaling is critical for controlling the regenerative capacity of mouse retinal ganglion and corticospinal neurons [5–6]. After development, the regrowth potential of retinal ganglion and corticospinal tract (CST) was lost and accompanied with down-regulation of mTOR in retinal ganglion cells and corticospinal neurons. Axonal injury further diminished neuronal mTOR activity in these neurons. Forced up-regulation of mTOR in retinal ganglion cells and corticospinal neurons by conditional deletion of PTEN enhanced axonal regeneration of injured optic nerve and CST [5–6].

A series of studies have shown that IL-6 can reactivate the intrinsic growth program of injured neurons and promote axonal regeneration, through modulating cAMP, JAK/STAT3 and/or PTEN/mTOR signaling pathways [7–10]. Under normal physiological conditions, levels of IL-6 remain low, however, IL-6 is elevated rapidly in the PNS after axotomy [11] and in the CNS after brain damage including ischemia [12] and trauma [13–16]. In the PNS, the neuropoietic cytokines and their major signaling pathways are activated in primary sensory neurons by axonal injury and are necessary for a full regenerative response in these neurons. In the CNS, exogenous IL-6 also promotes sprouting and functional recovery by reactivating intrinsic growth capacity of injured neurons [9–10]. Our previous study has shown that IL-6 stimulated the neurite outgrowth of DRG neurons cultured on a myelin substrate, by inducing the expression of regeneration-associated genes [10]. Furthermore, *in vivo* intrathecal delivery of IL-6 immediately after CST injury induced sprouting and increased the expression of mTOR in neurons around the lesion site, accompanied by improved functional recovery [10]. However, whether intrathecal delivery of IL-6 immediately after CST injury also reactivates the intrinsic growth program of these axotomized CST pyramidal cells in the sensorimotor cortex remains largely unknown.

In the current study, we analyzed the effects of intrathecal delivery of IL-6 on the expression of cAMP, adenylyl cyclase (AC) and phosphodiesterases (PDE) in the sensorimotor cortex by ELISA, on the expression of regeneration-associated genes by RT-PCR, and on the expression of pSTAT3 and mTOR, downstream IL-6/JAK/STAT3 and PI3K/AKT/mTOR signaling pathways respectively, by immunohistochemistry and Western blot. We showed that intrathecal delivery of IL-6 immediately after spinal cord injury elevated cAMP level in the sensorimotor cortex and increased the expression of growth-associated genes GAP-43, SPRR1A, CAP-23 and JUN-B and increased the expression of pSTAT3 and mTOR in the pyramidal cells of the sensorimotor cortex, layers V and VI. However, AG490, an inhibitor of JAK, partially blocked these effects of IL-6. All these results indicate that intrathecal delivery of IL-6 immediately after spinal cord injury can reactivate the intrinsic growth capacity of pyramidal cells in the sensorimotor cortex and the effects of IL-6 was associated with the activation of JAK/STAT3 signaling pathway.

## Materials and Methods

Adult female Sprague-Dawley (SD) rats (200–250g) were provided by the Biological Service Center of Third Military Medical University, Chongqing, China. All experiments were performed with the approval of the Ethics Committee of Third Military Medical University. Every effort was made to minimize the number of animals sacrificed and to limit animal suffering. The animals were housed in the plastic cages under a 12-h light/dark cycle. Food and water were available ad libitum.

### Intrathecal catheter

The rat intrathecal catheters were implanted as described previously [10]. Briefly, adult female SD rats (200g–250g) were anesthetized with 3.5% chloral hydrate (10 ml/kg, i.p.), the dorsal aspect of the L4-L5 vertebra of the rat was opened and the L5 spinous process was removed. A polyethylene catheter (PE-10; gift from Professor XF Zhou, Flinders University, Australia) was implanted about 1.0 cm through the incision between the L4-L5 vertebra interval. The incision was sutured in layers, another end of the catheter was tunneled subcutaneously and exited from the neck of the rat and secured in situ. Correct intrathecal placement was confirmed by the dragging or paralysis of bilateral hind limbs after injection of 2% lidocaine (10 μl) through the catheter. The rats were allowed to recover for 3–5 days before dorsal hemisection of the spinal cord was performed.

### Corticospinal tract transection and Fluorogold retrograde labeling pyramidal cells in the sensorimotor cortex layer V and VI

Above cathetered rats were anesthetized with 3.5% chloral hydrate (10 ml/kg, i.p.), and underwent laminectomy at vertebral level T9/10 to expose the spinal cord. Before transection of the spinal cord, retrograde neuronal tracer Fluorogold (FG, 2% in PBS, Invitrogen, 2 μl per each side in four points) was injected into the left and the right dorsal column to label pyramidal cells in the sensorimotor cortex layer V and VI. And then the dorsal half of the spinal cord was cut with a pair of previously marked microscissors to sever the dorsal corticospinal tract (CST) at a depth of 1.8 mm from the dorsal surface, and a 25 gauge needle was used to plough the lesion site to assure complete transection of the CST. Muscles and skin were closed in layers, and animals were placed in a temperature-controlled chamber until thermoregulation was re-established. Histological examination had revealed that these lesions severed all dorsal CST fibers in the dorsal funiculus as well as in the lateral CST and extended past the central canal in all animals. Manual voiding of the bladder was performed twice per day until reflex bladder emptying was reestablished.

### Intrathecal delivery of IL-6

For IL-6 treatment group (n = 10), 10 μl (20 ng/10μl) recombinant rat IL-6 (PeproTech) and then 15 μl saline was intrathecally delivered. For IL-6 and AG490 group (n = 10), 10 μl (20 ng/10μl) recombinant rat IL-6 and then 5μl (1 μM/5μl) AG490 (Cayman), an inhibitor of JAK, and then 10 μl saline was intrathecally delivered. And for the control (saline) group (n = 10), 25 μl saline was intrathecally delivered. The delivery was performed daily immediately after CST transection, and lasted for 7 days.

### RNA extraction, reverse transcription and PCR

To observe the expression of growth-associated genes GAP-43, CAP-23, SPRR 1A, IL-6, JUN B and c-JUN after intratheral delivery of IL-6, total RNA was extracted from both sides of the sensorimotor cortex (around 2 mm posterior from the bregma, 2 mm lateral from the midline, with a depth of 1.5 mm from the dura matter of the brain) [10, 17] with Trizol (Invitrogen) 7 days after delivery of IL-6, or IL-6 and AG 490, or saline (n = 3, respectively). Total RNA (100 ng) was reverse transcribed and amplified using TaqMan One-Step RT-PCR Master Mix (Applied Biosystems, Foster City, CA). Gene expression of β-actin was used for internal control. Bands intensities were analyzed by Image J analysis software. Results were normalized to β-actin and were expressed relative to data of saline group, which was arbitrarily assigned a value of 1. Primers were synthesized by BioFlux company (Table 1).

**Table 1:** List of primers for RT-PCR.

Name	forward	reverse	Anneal T (°C)	cycles
**GAP-43**	5’-AGCCAAGGAGGAGCCTAAAC-3’	5’-CTGTCGGGCACTTTCCTTAG-3’	56°C	35
**SPRR 1A**	5’-TCCATCACCATACCAGCAGA-3’	5’-TAGCACAAGGCAATGGGACT-3’	56°C	35
**CAP23**	5’-ACCCAGAAGGAGAGCGAAC-3’	5’-GTCGGCCTCCTTTTCCTC-3’	57°C	35
**Jun-B**	5’-GCCTCCGGGACAGTACTTTTA-3’	5’-CGTCACGTGGTTCATCTTG-3’	56°C	35
**C-Jun**	5’-ACCACTTGCCCCAACAGAT-3’	5’-CTTGATCCGCTCCTGAGACT-3’	56°C	35

### Immunohistochemistry

Immunohistochemical staining was performed on fixed brain or spinal cord. Animals were killed 7 days after intratheral delivery of IL-6, IL-6 and AG490 or saline (n = 4 respectively), by transcardial perfusion with 0.9% saline followed by 4% paraformaldehyde in 0.1 M phosphate buffer (PBS). The brain and 10 mm spinal cord centered at the lesion site were dissected, postfixed in the same fixatives, and soaked in 30% sucrose solution. 30 μm thickness of cryostat sections of the brain through the sensorimotor cortex or the spinal cord were cut in coronary section. Sections were blocked in TBS with 5% normal donkey and 5% normal goat serum for 1 h and then incubated with primary antibodies overnight at 4°C. Fluorescence conjugated secondary antibodies were applied for 2 h at room temperature. The primary antibodies were omitted in the primary antibody negative control group. The following primary antibodies were used: mouse anti-βIII tubulin antibody (1:400, Sigma), rabbit anti-gp130 (1:200, Bioworld), rabbit anti-pSTAT3 (S727) antibody (1:100; Bioworld), rabbit anti-mTOR antibody (1:500; Cell Signaling). Images were analyzed with Image J software to measure the fluorescence intensity by drawing around cell bodies of mTOR or pSTAT3 positive neurons after deduction the background signal. Each group 100 neurons were analyzed.

### Western blot

Seven days after spinal cord injury and intrathecal delivery of IL-6, IL-6 and AG490, or saline (n = 3 respectively), both sides of the sensorimotor cortex (around 2 mm posterior from the bregma, 2 mm lateral from the midline, with a depth of 1.5 mm from the dura matter of the brain) [10, 17] were collected in cold PBS then homogenized in tissue lysis buffer containing 20 mM Tris-HCl, 150 mM NaCl, 2 mM EDTA, 0.1 mM EGTA, 1% Triton X-100, and 0.5% deoxyocholine with complete protease inhibitors (Roche). Samples were kept on ice for 30 min and then centrifuged at 13,000 g for 15 min to remove cell debris. The protein concentration in the supernatant was measured with the Bradford assay using BSA as a standard. Sixty micrograms of proteins were subjected to 10% sodium dodecyl sulfate polyacrylamide gel electrophoresis using dual color protein standards (Bio-Rad) as markers. After protein transfer, the polyvinylidene difluoride membrane was blocked with 5% non-fat milk in Tris-buffered saline with 0.1% Tween-20 for 1 h. Membranes were probed with primary antibodies overnight at 4°C and a peroxidase-conjugated secondary antibody was applied for 2 h at room temperature. Immunosignals were detected using the ECL Plus kit (GE Healthcare, Chalfont St Giles, United Kingdom). Primary antibodies used included rabbit anti-ERK1/2 (1:200, Cell Signaling), rabbit anti-pERK1/2 (Thr202/Tyr204) (1:200, Cell Signaling), rabbit anti-pSTAT3 antibody (1:100; Bioworld), rabbit anti-mTOR antibody (1:500; Cell Signaling), and mouse anti-β-actin antibody (1:500; Cell Signaling) which was used as an internal control for quantification of blots. Blots intensities were analyzed by Image J analysis software. Results were normalized to β-actin and were expressed relative to data of saline group, which were arbitrarily assigned as 1.

### ELISA for cAMP, AC and PDE

Thirty microgram of above protein were subjected to measurement of cAMP, AC and PDE by using commercially available ELISA kits (ENZO, ADI-900-067; SBIO, DRE30702; SBIO, DRE30704 respectively). Results are expressed as percentage of maximum.

### Statistics

The data are expressed as the mean ± standard error of the mean (SEM). Statistical analysis was performed by using Student *t* test and one way ANOVA (analysis of variance) with post hoc comparisons and Bonferroni correction on SPSS software. Differences were considered to be significant when P < 0.05.

## Results

### Intrathecal delivery of IL-6 increases cAMP levels in the sensorimotor cortex

**Fig 1:**
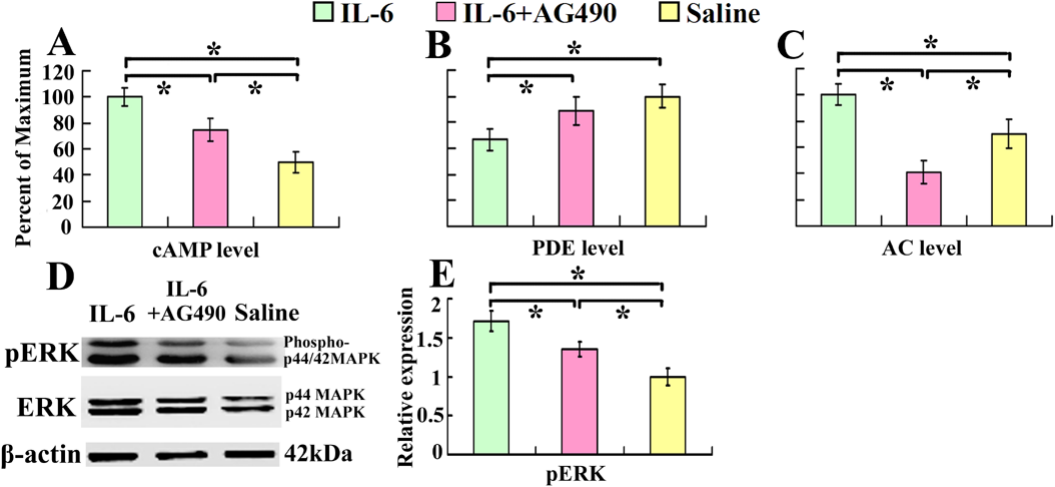
Intrathecal delivery of IL-6 increases cAMP and AC levels and decreases PDE level in the sensorimotor cortex. Seven days after intrathecal delivery of IL-6, IL-6 and AG-490, or saline, cAMP, AC and PDE levels in the sensorimotor cortex were determined by ELISA. cAMP (A) and AC (C) levels increased while PDE (B) level decreased in IL-6 delivery group compared with that of saline group. After treatment with AG490, an inhibitor of JAK, the effects of IL-6 were partially blocked. Data were expressed as percentage of maximum (means ± SEM; n = 3). *P < 0.01. D and E demonstrate that IL-6 activates ERK. The expression of pERK was increased in IL-6 delivery group compared with that of saline group, which was also partially blocked by AG-490. Data were expressed as relative density of that of saline delivery after normalized to β-actin (means ± SEM; n = 3). *P < 0.01.

It has been well documented that cAMP is associated with intrinsic growth capability of neurons. cAMP degradation by PDE can be inhibited through activation of ERK [18]. We observed that intrathecal delivery of recombinant rat IL-6 for 7 days, immediately after spinal cord injury, increased cAMP level (Fig 1A), and decreased PDE level (Fig 1B) respectively in the sensorimotor cortex. At the same time pERK increased 1.67 times compared with that of saline control (Fig 1D and 1E). We also demonstrated that AC level was increased after IL-6 treatment (Fig 1C). To explore the relevant mechanisms, we introduced AG490, an inhibitor of JAK, at the same time. As expected, AG490 partially blocked the IL-6-induced elevation of the cAMP level (Fig 1A).

### Intrathecal delivery of IL-6 increases the expression of regeneration-associated genes in the sensorimotor cortex

**Fig 2:**
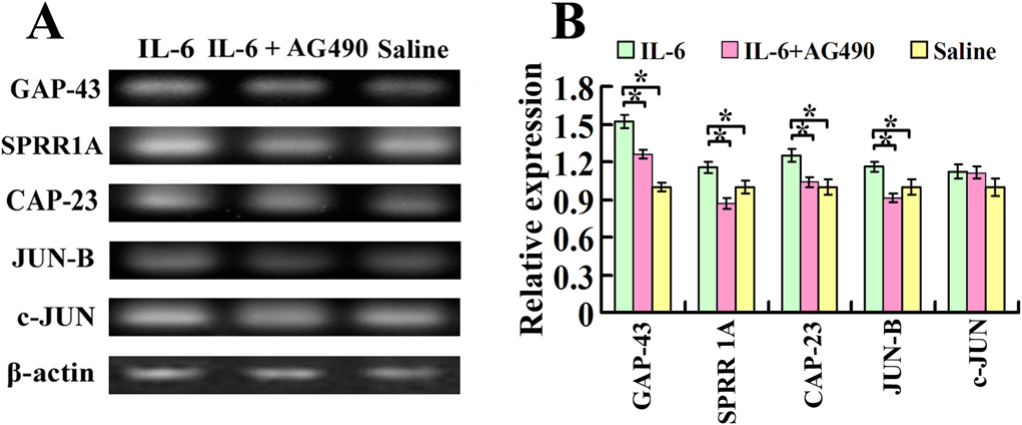
Expression of growth-associated genes in the sensorimotor cortex after intrathecal delivery of IL-6. A. Seven days after spinal cord injury and intrathecal delivery of either IL-6, IL-6 and AG490, or saline, mRNAs for GAP-43, SPRR1A, CAP-23, JUN-B and c-JUN were measured by RT-PCR. IL-6 increased the expression of mRNAs for GAP-43, SPRR1A, CAP-23 and JUN-B in comparison to that of saline delivery. While c-JUN did not show significant difference among these three groups. B. Quantification of gene expression using densitometry. Data were expressed as relative expression of saline group after normalized to β-actin (means ± SEM; n = 3). *P < 0.01.

Seven days after spinal cord injury and intrathecal delivery of recombinant rat IL-6, IL-6 and AG490, or saline, total mRNA was prepared from both sides of the sensorimotor cortex for the RT-PCR assay. Quantitative analysis of the optical density observed in three independent experiments illustrated that the expression of mRNAs for GAP-43, SPRR1A, CAP-23 and JUN B increased significantly in the sensorimotor cortex of IL-6 group, compared to saline group (Fig 2A and 2B). AG490 treatment partially blocked IL-6-induced upregulation of GAP-43 and CAP-23 and completely blocked IL-6-induced upregulation of SPRR1A and JUN B (Fig 2A and 2B), while c-JUN did not show significant difference among these three groups (Fig 2A and 2B).

### gp-130 receptor is expressed by both pyramidal and spinal cord neurons

**Fig 3:**
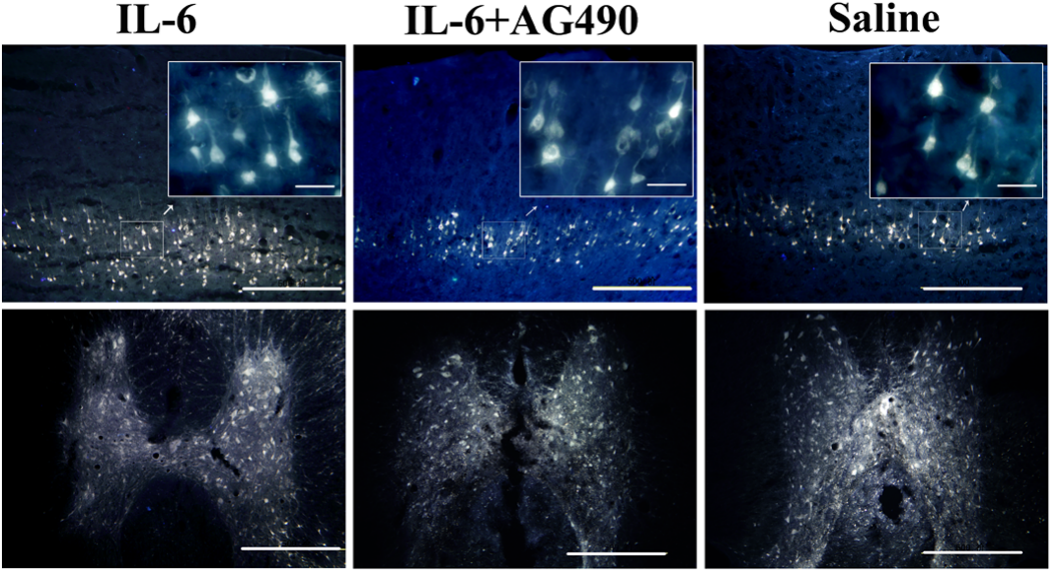
Expression of Fluoroglod (FG) in the sensorimotor cortex and the spinal cord. Both pyramidal cells in the V and VI layers of the sensorimotor cortex (upper panel) and neurons in the spinal cord gray matter (lower panel) contain FG (Scale bar: 500 μm). Insets of upper panel show the higher magnification of FG-labelled pyramidal cells in selected areas of the sensorimotor cortex layers V and VI. Scale bar: 50 μm.

**Fig 4:**
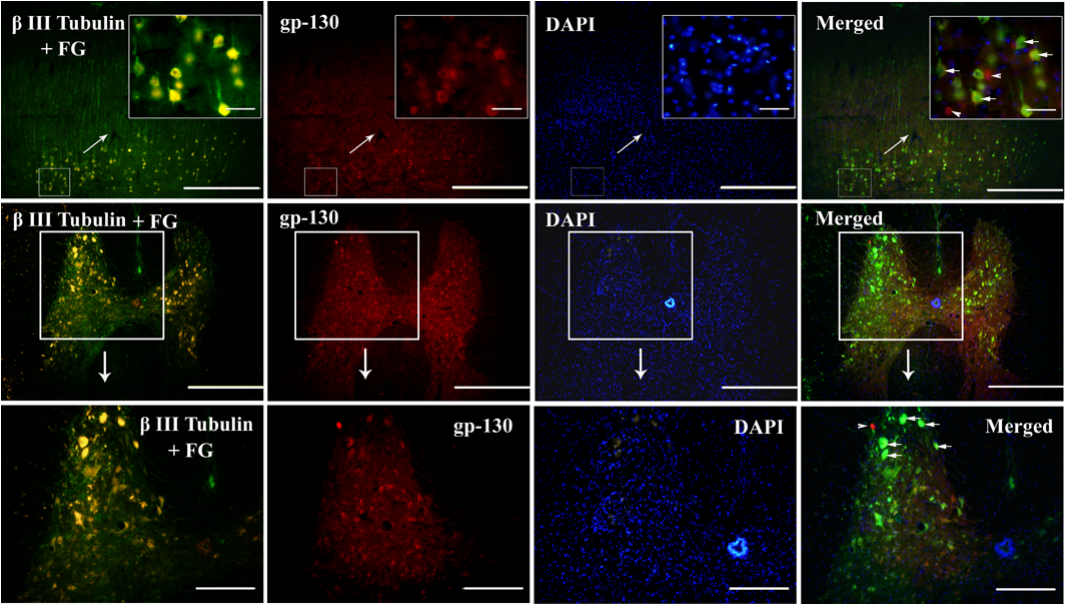
Expression of gp-130 in the sensorimotor cortex and the spinal cord. Pyramidal cells were double labeled with FG and βIII- tubulin. Both pyramidal cells in the sensorimotor cortex layers V and VI (upper panel) and neurons in the spinal cord gray matter (middle and lower panel) showed gp-130 positive staining. Scale bar: 500 μm for upper panel and middle panel and 200 μm for lower panel. Insets of upper panel show the higher magnification of gp-130-labelled pyramidal cells in selected areas of the sensorimotor cortex layers V and VI. Scale bar: 50 μm. Lower panel shows the higher magnification of neurons in the middle panel. Scale bar: 200 μm. Arrowheads indicate cells are both fluorogold and tubulin labeled in the sensorimotor cortex and spinal cord, whereas arrows point to tubulin labeled cells.

To investigate whether IL-6 might exert its growth effect through the common signal transducer, namely gp130 (glycoprotein 130, also known as CD130) of which all the IL-6 family members share [19–22], we examined the expression of gp-130 in pyramidal cells of the sensorimotor cortex layers V and VI and the spinal cord neurons. Firstly we injected retrograde neuronal tracer FG into the dorsal column of the spinal cord to label pyramidal cells in the sensorimortex cortex layers V and VI (Fig 3), combined with βⅢ-tubulin immunostaining. We showed that immunoreactivity of gp-130 was colocalized with that of FG and βⅢ-tubulin in pyramidal cells in the sensorimotor cortex (Fig 4) and in neurons of the spinal cord (Fig 4).

### Intrathecal delivery of IL-6 activates JAK/STAT3 signal pathway in pyramidal cells of the sensorimotor cortex after spinal cord injury

**Fig 5:**
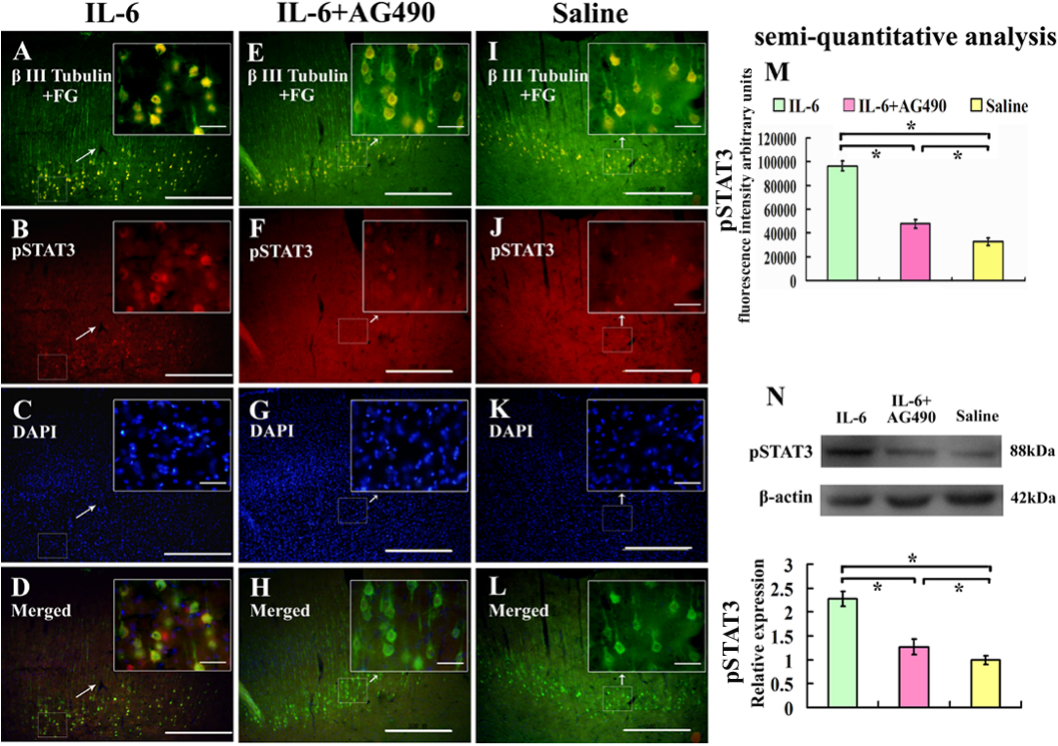
Effect of IL-6 on the expression of pSTAT3 in the sensorimotor cortex. Pyramidal cells in the sensorimortex cortex were labelled with FG and βIII tubulin immunostaining. IL-6 delivery (A-D) increased the expression of pSTAT3 compared with that of saline treatment (I-L), and AG490 partially blocked this effect of IL-6 (E-H). These results were further confirmed by quantification of fluorescence intensity (M) and semiquantitative analysis by Western blot (N). Lower panel of the N shows the quantification of pSTAT3 expression using densitometry. Data were expressed as relative expression of that of saline group after normalized to β-actin (means ± SEM; n = 3). *P < 0.01. Scale bar: 500 μm. Insets show the higher magnification of pyramidal cells in selected areas of the sensorimotor cortex V and VI layers. Scale bar: 50 μm.

IL-6 activated the JAK/STAT3 signal pathway manifested by increased pSTAT3 immunoreactivity in pyramidal cells of the sensorimotor cortex (Fig 5A–5D) compared with that of saline group (Fig 5I–5L). Quantification of fluorescence intensity of pSTAT3 in FG and βⅢ- tubulin labeled pyramidal cells (Fig 5M) and semiquantitative analysis of pSTAT3 protein by Western blot analysis (Fig 5N) further confirmed increased pSTAT3 level in the sensorimotor cortex of IL-6 group compared with that of saline group (Fig 5M and 5N). AG490, an inhibitor of JAK, partially blocked IL-6-induced increase of pSTAT3 (Fig 5E–5H, 5M and 5N).

### Intrathecal delivery of IL-6 activates PI3K/AKT/mTOR signal pathway in pyramidal cells of the sensorimotor cortex after spinal cord injury

**Fig 6:**
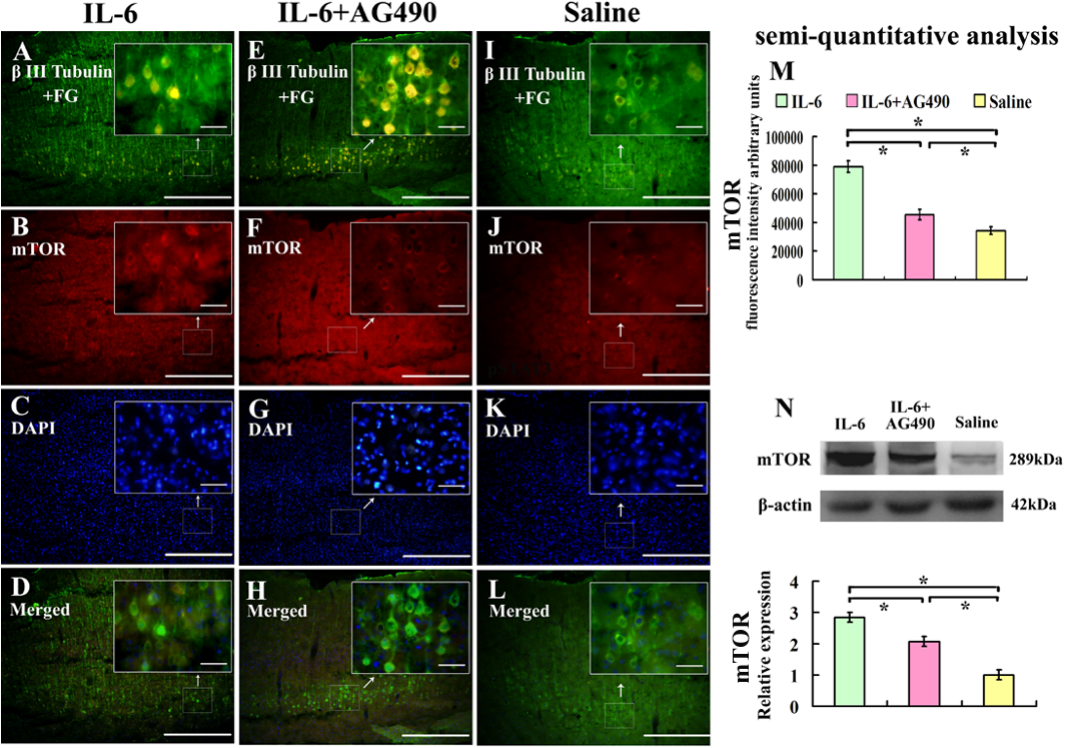
Effect of IL-6 on the expression of mTOR in the sensorimotor cortex. Pyramidal cells in the sensorimortex cortex were labelled with FG label and β-III tubulin immunostaining. IL-6 delivery increased the expression of mTOR (A-D) compared with that of saline treatment (I-L), and AG490 partially blocked this effect of IL-6 (E-H). These results were further confirmed by quantification of fluorescence intensity (M) and semiquantitative analysis by Western blot (N). Lower panel of the N shows the quantification of mTOR expression using densitometry. Data were expressed as relative expression of that of saline treatment after normalized to β-actin (means ± SEM; n = 3). *P < 0.01. Scale bar: 500 μm. Insets show the higher magnification of the pyramidal cells in selected areas of the sensorimotor cortex layers V and VI. Scale bar: 50 μm.

To test whether intrathecal delivery of IL-6 also activated PI3K/AKT/mTOR signal pathway in pyramidal cells in the sensorimotor cortex, we investigated mTOR expression in pyramidal cells in the sensorimotor cortex layers V and VI by immunostaining and Western blot. We showed that immunoreactivity of mTOR was upregulated in FG and βIII- tubulin labeled pyramidal cells in the sensorimotor cortex, layers V and VI (Fig 6A–6D and 6M), which was in line with Western blot analysis (Fig 6I–6L and 6N). AG490 partially blocked IL-6-induced up-regulation of mTOR (Fig 6E–6H, 6M and 6N).

## Discussion

We have previously shown that IL-6 promotes axonal regeneration of axotomized CST and functional recovery by enhancing synapse formation and increasing the expression of mTOR in neurons around the lesion site [10]. In this study, we further delineate that intrathecal delivery of IL-6 immediately after spinal cord injury induces pronounced up-regulation of cAMP level and regeneration-associated genes GAP-43, SPRR1A, CAP-23 and JUN-B and activated not only JAK/STAT3 signaling pathway but also PI3K/AKT/mTOR and ERK signaling pathways in pyramidal cells of the sensorimotor cortex.

**Fig 7:**
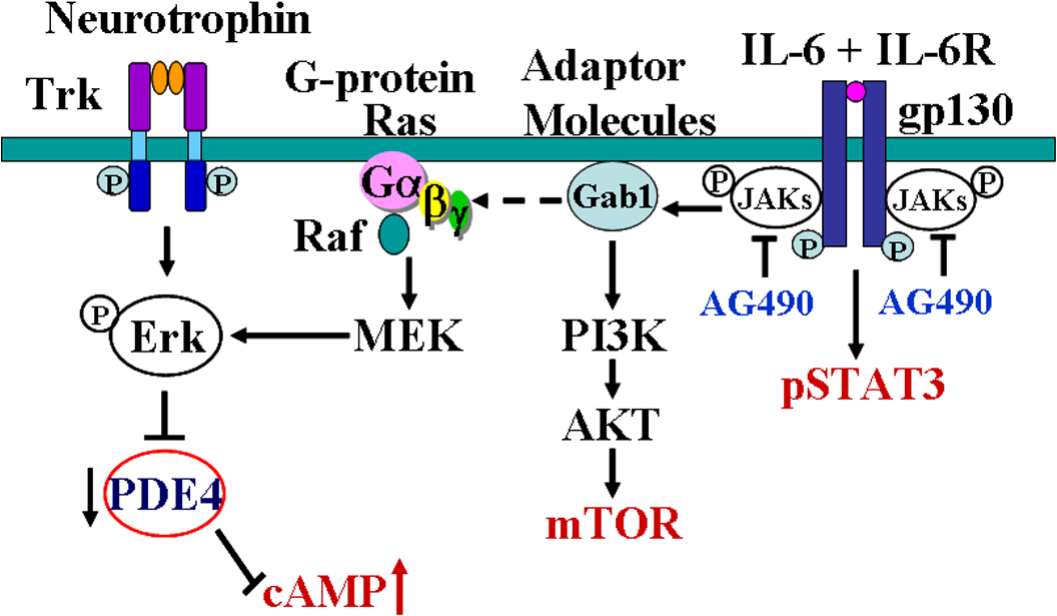
Diagram shows the possible mechanisms of IL-6 in regulating cAMP level in neurons. Binding of neurotrophins to the corresponding receptors (Trk) activates ERK which inhibits PDE and then inhibits the degradation of cAMP [18]. IL-6 binds to IL-6R causing dimerization of gpl30, thereby activating the intracellular tyrosine kinases of the Janus kinase family (JAKs) and subsequently activating STAT3. In the other hand, IL-6 modulates ras and raf through an adapter molecule Gab1, leading to the activation of threonine kinase of MAP kinase family, which include ERK [20–21]. IL-6 activates ERK, thus inhibits PDE and then inhibits the degradation of cAMP. IL-6 may also activate PI3K/Akt pathway [19], through which IL-6 induces mTOR expression in neurons.

The diminished intrinsic growth capacity in CNS neurons is closely associated with the decline of cAMP during development [1]. After spinal cord injury, levels of cAMP in neurons of the brainstem and sensorimotor cortex decrease substantially, especially in their severed axon ends[4]. Manipulations aiming to increase levels of cAMP have been shown to facilitate survival and axonal regeneration of adult mammalian CNS neurons and promote functional recovery [4]. It has been reported that one of the molecular mechanisms underlying neurotrophin promoted neurite outgrowth is by increasing cAMP level through activating extracellular signal-regulated kinase (ERK) [18, 23–24], which inhibits the degradation of cAMP by PDE [18, 24](Fig 7). In present study we found that intrathecal delivery IL-6 immediately after CST injury increased cAMP level and decreased PDE level in the sensorimoter cortex and at the same time activated ERK (Fig 1). Multiple studies demonstrated that IL-6 facilitates neurite outgrowth by counteracting the release and/or the effects of TNF-α by inhibiting the synthesis of TNF-α in astrocytes [25–28] and facilitate astrocytes phagocytic activity to remove myelin debris thus enhance sprouting [29–30]. Furthermore, IL-6 might modulate the synthesis and release of multiple neurotrophic factors that are involved in the axonal regeneration after injury, such as NGF by astrocytes [16] or BDNF and GDNF by microglia [31]. It is reported that NGF binds to the TrkA receptor on the distal axon and activates endocytosis and retrograde transport of a ‘signaling endosome’ containing NGF bound TrkA, and then activates Erk1/2, p38, and Akt [32–34]. And the retrograde transport of Trk receptor complex uses a microtubule-based retrograde transport protein dyenin as a motor [35]. In recent study German et al evaluated the localization of STAT3 and Erk1/2 from IL-6 treated cells and demonstrated that a retrograde transport protein dynein was highly enriched within 115,000 ×g fraction, suggesting the presence of mobile, vesicular structures [22]. It is possible that intrathecal delivered IL-6 acts through similar retrograde transport mechanism to increase cAMP and decreased PDE in the sensorimoter cortex. However, it has been demonstrated that in the PNS IL-6 is not retrogradely transported from distal axon to neuron body [36], thus rather than retrogradely transported itself, IL-6 may trigger the retrograde transport of a variety of other signaling molecules such as NGF, STAT3. Whereas, multiple studies demonstrated that signal transduction over large distances, as exists between the distal axon and cell body in neurons, often employ endocytic structures to properly target and transport signals across vast intracellular space [34, 37]. German et al recently demonstrated that IL-6/STAT3 signaling system uses endosomes in a fundamentally different manner than other receptor tyrosine kinase signaling systems [22]. IL-6 binds to its specific receptor IL-6R causing dimerization of the integral signaling protein gp130 upon ligand binding [20–21, 38]. gp130 dimerization induces receptor complex internalization into an endosomal structure. These endosomes then function as beacons of activation as they move through the endocytic pathway, directly and catalytically phosphorylating local cytoplasmic populations of STAT3 through transient interactions [22]. MAPK signaling is initiated by the recruitment of Shc and SHP-2 to the cytoplasmic tail of gp130 following IL-6 treatment [38–40]. Apart from signal transport, these endosomes are associated with the regulation of spatial and temporal dynamics of signal initiation, propagation and cessation [41]. STAT3 and MAPK have both been localized to endosomal structures and are dependent upon endocytosis for downstream signaling activation, and crosstalk between MAPK and STAT3 signaling systems is a requisite for maximal STAT3 transcriptional activity, and these events are depend upon Erk1/2 activation from late endosomal structures [22]. In this study we did observe that increased expression of pERK (Fig 1D) after IL-6 treatment, which is in parallel with decreased expression of PDE (Fig 1B) and elevation of cAMP (Fig 1A). It is possible that intrathecal delivery IL-6 immediately after CST injury reactivate the intrinsic growth capacity in sensorimotor cortex through these IL-6R containing endosomal structures (Fig 7). Also as the coherence in the expression of gp-130 staining in both the sensorimotor cortex and in the spinal cord grey matter, it is possible that activated neurons or glia in the spinal cord grey matter may produce some unknown retrograde signals to change the biochemistry of CST neurons.Furthermore, we observed that intrathecal delivery of IL-6 immediately after spinal cord injury also elevated mTOR in sensorimotor cortex. It has been reported that mTOR pathway play an important role in determining the intrinsic axon regrowth responsiveness of injured CNS neurons[6]. Activation of the mTOR pathway in RGCs or corticospinal neurons enhanced their axonal regeneration [5–6]. Inflammation or treatment with CNTF/LIF activated mTOR activity in retinal ganglion cells (RGCs) and facilitated long distance regeneration of RGCs after axotomy [42]. IL-6 has also been shown to activate PI3K/Akt pathway [19]. Therefore, mTOR is one possible signaling molecule that IL-6 acts through to enhance axonal regeneration of axotomized pyramidal cells (Fig 7).

In conclusion, we further showed that intrathecal delivery of IL-6 immediately after spinal cord injury elevated cAMP level and up-regulated the expression of regeneration-associated genes including GAP-43, SPRR1A, CAP-23 and JUN-B, and the expression of pSTAT3 and mTOR in pyramidal cells of the sensorimotor cortex. It is possible that IL-6 acts through ‘signaling endosome’ and is associated with the activation of JAK/STAT3 signaling pathway.
